# Innovative Approach in the Cryogenic Freezing Medium for Mesenchymal Stem Cells

**DOI:** 10.3390/biom12050610

**Published:** 2022-04-20

**Authors:** Nela Pilbauerova, Jan Schmidt, Tomas Soukup, Tomas Prat, Kristina Nesporova, Vladimir Velebny, Jakub Suchanek

**Affiliations:** 1 Department of Dentistry, Charles University, Faculty of Medicine in Hradec Kralove and University Hospital Hradec Kralove, Sokolska 581, 50005 Hradec Kralove, Czech Republic; nela.pilbauerova@lfhk.cuni.cz (N.P.); jan.schmidt@lfhk.cuni.cz (J.S.); suchanekj@lfhk.cuni.cz (J.S.); 2 Department of Histology and Embryology, Faculty of Medicine in Hradec Kralove, Charles University, Simkova 870, 50003 Hradec Kralove, Czech Republic; soukupto@lfhk.cuni.cz; 3 Contipro a.s., Dolni Dobrouc 401, 56102 Dolni Dobrouc, Czech Republic; kristina.nesporova@contipro.com (K.N.); vladimir.velebny@contipro.com (V.V.)

**Keywords:** hyaluronic acid, dental pulp stem cells, adipose tissue-derived stem cells, cryopreservation, cryoprotective agent, human mesenchymal stem cells

## Abstract

The physical stresses during cryopreservation affect stem cell survival and further proliferation. To minimize or prevent cryoinjury, cryoprotective agents (CPAs) are indispensable. Despite the widespread use of 10% dimethyl sulfoxide (DMSO), there are concerns about its potential adverse effects. To bypass those effects, combinations of CPAs have been investigated. This study aimed to verify whether high-molecular-hyaluronic acid (HMW-HA) serves as a cryoprotectant when preserving human mesenchymal stem cells (hMSCs) to reduce the DMSO concentration in the cryopreservation medium. We studied how 0.1% or 0.2% HMW-HA combined with reduced DMSO concentrations (from 10% to 5%, and 3%) affected total cell count, viability, immunophenotype, and differentiation potential post-cryopreservation. Immediately after cell revival, the highest total cell count was observed in 10% DMSO-stored hMSC. However, two weeks after cell cultivation an increased cell count was seen in the HMW-HA-stored groups along with a continued increase in hMSCs stored using 3% DMSO and 0.1% HMW-HA. The increased total cell count corresponded to elevated expression of stemness marker CD49f. The HA-supplemented cryomedium did not affect the differential potential of hMSC. Our results will participate in producing a ready-to-use product for cryopreservation of mesenchymal stem cells.

## 1. Introduction

Over the past decades, dental tissues have become an attractive source of adult human mesenchymal stem cells (hMSCs). Dental pulp stem cells (DPSCs) were first identified by Gronthos in 2000 [1], and since then an increasing amount of research has pointed out the broad spectrum of multipotency capacity and potential applicability that DPSCs have in treating numerous disorders, such as endocrine disorders, neurodegenerative diseases, spinal injury, and peripheral nerve injury, and in repairing and regenerating bones and cartilage [2]. Easy harvesting from extracted teeth together with a wide range of multilineage differentiation capacity makes these stem cells a suitable alternative for adult mesenchymal stem cells isolated from bone marrow. Importantly, DPSCs appear to retain their proliferation capacity, phenotype, and multipotency after cryogenic storage at sub-zero temperatures [3,4,5,6,7,8], which is a key aspect in their long-term preservation.

In this rapidly growing field, a vast number of freezing protocols have been tested for the cryopreservation of DPSCs. Cryopreservation and freezing conditions represent an insult to stem cell metabolism. It is essential to implement protocols that minimize potential cell cryoinjuries and facilitate a high rate of stem cell post-thaw recovery. In general, cryopreservation, namely for cell therapy, brings a lot of pitfalls, including cooling rate, the use of animal-derived culture medium additives, and the potential cytotoxicity of cryoprotective agents (CPAs). Only through assessment of these challenges and implementation of standardized good manufacturing practices (GMP) will stem cell treatment options improve. When cells are cooled too slowly, they dehydrate and shrink due to osmotic stress, whereas rapid cooling results in intracellular ice formation [9,10,11]. Cell membranes were identified by Rowe [12] as one of the key targets for freezing injury. Besides carefully controlling the optimal cooling rates, the medium can also be supplemented with CPAs in order to minimize cryoinjury.

In general, CPAs can be divided into two different groups: low- and high-molecular-weight CPAs [11,13]. The low-molecular-weight CPAs include dimethyl sulfoxide (DMSO), glycerol, and ethylene (propylene) glycerol. These CPAs can penetrate the cell cytoplasmic membrane, preventing ice crystal nuclei formation and slowing down the rate of ice crystal growth inside cells [9,14]. The ability of CPAs to form hydrogen bonds with water was proposed as a strong correlate with their protective ability [15]. However, their concentrations must stay below the threshold for cell toxicity [15]. The second group, high-molecular-weight CPAs (HMW-CPAs), includes dextran, hydroxyethyl starch, polyvinylpyrrolidone, and polyvinyl alcohol. These HMW-CPAs remain in the extracellular space and participate in cell dehydration; thus minimizing intracellular ice crystal formation and helping in membrane stabilization [11].

The most versatile and commonly used CPA is DMSO at a concentration of 10%. Woods et al. reported that 7.8–11.6% DMSO was optimal for DPSCs cooled at −1 °C/min in an isopropanol bath of up to −85 °C for 24 h followed by storage in liquid nitrogen [8]. Despite the widespread use of DMSO, there are also concerns about its potential cytotoxic effect. DMSO-preserved bone marrow cells have been shown to cause adverse effects after their transplantation [16,17]. Moreover, DMSO-related influence on gene expression of gene methylation machinery was observed and thus represents a major obstacle of DMSO usage in, e.g., alternative stem cell sources [18]. Therefore, many research strategies have been exploring ways to reduce cryoprotectant toxicity and at the same time minimize stem cell cryoinjuries. Hoping to lower or even completely remove DMSO from the freezing solutions, several other compounds have been investigated. Given the multiplicity of actions of CPAs and the need to mitigate toxicity, it was hypothesized that combinations of CPAs might be more effective [15]. CPA combinations were developed by mixing both permeating and non-permeating CPAs, a philosophy that has been successfully used in many CPA protocols [15]. The aim is to find a balance between extracellular and intracellular cell protection during freezing conditions and thereby gain a high number of functional and vital stem cells after thawing.

In many recent studies, these combinations have allowed DMSO concentrations to be lowered to 5% while maintaining rates of stem cell cryopreservation comparable to 10% DMSO concentrations [11,19,20]. However, freezing media containing CPAs in combination with DMSO concentrations of less than 2% have resulted in lower effectivity rates in post-thawing functional stem cells [21]. Using a freezing protocol involving a magnetic field, DPSCs can be frozen in a serum-free freezing solution containing only 3% DMSO [22]. However, cryopreservation protocols involving the magnetic field are technically and financially demanding. We had already verified that the less technically and financially demanding technique, i.e., uncontrolled-rate freezing, is effective in the cryopreservation of DPSCs [3]. Therefore, we focused this study on lowering the DMSO concentration by mixing it with a non-penetrating CPA. Hyaluronic acid (HA) is an acidic, non-sulfated glycosaminoglycan with a repeating disaccharide structure of d-glucuronic acid and N-acetyl-d-glucosamine [23]. It is one of the main components of the extracellular matrix, and in high molecular form, it is part of the cell’s native environment. Furthermore, the chemical and bioactive properties of HA, such as the ability to bind water molecules or form a framework, provide properties that can be beneficial during stem cell cryopreservation.

The first research question was whether HMW-HA (>1.0 MDa) can be used as a cryoprotectant when preserving DPSCs in order to reduce the concentration of DMSO in the cryopreservation medium. We then sought to identify the optimal combination of HMW-HA and reduced-dose DMSO. Several studies had already identified HA as a non-toxic material that does not negatively affect the viability, proliferation activity, or differentiation potential of DPSCs [24,25]. The second question was whether DPSCs stored using the combination of HMW-HA and reduced-dose DMSO could withstand freezing conditions and maintain their stemness markers and multipotency capacity after thawing. Finally, we wanted to verify whether the innovative approach in the cryogenic freezing medium can be used in cryostorage of stem cells other than dental pulp stem cells.

## 2. Materials and Methods

### 2.1. hMSC Lineages

All human DPSC lineages used in this study had been previously isolated and characterized according to their phenotype and differentiation potential. Results had already been published in our previous study [3]. Briefly, permanent tooth extraction occurred at the Dental Clinic of the University Hospital Hradec Kralove, Czech Republic. Patients or their legally authorized representatives voluntarily signed written informed consent before being involved in the study. The Ethical Committee in Hradec Kralove, Czech Republic, approved the guidelines for this study (ref. no. 201812 S07P). We isolated DPSCs by means of an enzymatic isolation technique using a 0.05% trypsin/EDTA solution (Gibco, London, UK).

Adipose-derived stem cells (hADSCs) were isolated from the stromal vascular fraction of lipoaspirate after an aesthetic liposuction surgery at CHS Galen, v.o.s.; Usti and Orlici; Czech Republic. All donors voluntarily signed written informed consent for the discarded material to be used for cell harvesting before being involved in the study. We isolated ADSCs using stromal tissue Collagenase I enzymatic digestion (0.3 U/mL (Wünsch units) as it was published earlier [26] with minor modifications.

### 2.2. hMSC Characterization

We used five independent human lineages of DPSCs cryopreserved in the 4th passage; for the cryopreservation process, we used uncontrolled-rate freezing and dimethyl sulfoxide (Sigma-Aldrich—Merck KGaA, Darmstadt, Germany) at a concentration of 10%. The DPSCs were stored at −80 °C. At the beginning of the study, we thawed lineages using a 37 °C thermal bath. Thawed DPSCs were seeded on an adherent tissue culture dish (TPP, Sigma-Aldrich) and cultivated in a modified cultivation medium (α-MEM, Gibco, Thermo Fisher Scientific, Foster City, CA, USA) for mesenchymal adult progenitor cells, containing 2% fetal bovine serum (FBS, PAA Laboratories, Dartmouth, MA, USA) and supplemented with 10 ng/mL epidermal growth factor (PeproTech, London, UK), 10 ng/mL platelet-derived growth factor (PeproTech), 50 nM dexamethasone (Bieffe Medital, Grosotto, Italy), 0.2 mM L-ascorbic acid (Bieffe Medital), 2% glutamine (Invitrogen, Carlsbad, CA, USA), 100 U/mL penicillin, 100 µg/mL streptomycin (Invitrogen), 20 μg/mL gentamicin (Invitrogen), and 2.5 µg/mL amphotericin (Sigma-Aldrich). The medium was also enriched with 10 μL/mL Insulin-Transferrin-Selenium-Sodium supplement (ITS, Bieffe Medital) to increase nutrient utilization. We incubated adherent tissue culture dishes (TPP, Sigma-Aldrich) with stem cells at 37 °C and 5% CO_2_ for 14 days to eliminate the effect of DMSO on previously stored cells that used DMSO as the CPA. We changed the cultivation medium every three days and passaged them once during this period, which was approximately after one week. Then, we reseeded stem cells in a concentration of 5000 cells per cm^2^ of an adherent tissue culture dish and cultivated them for another week. After 14 days, we measured the total cell count using a Z2-Counter (Beckman Coulter, Miami, FL, USA) and assessed the cell viability using a trypan dye exclusion method along with a Vi-Cell analyzer (Beckman Coulter).

Five independent hADSC lines were characterized according to their hMSC phenotype (CD73^+^; CD90^+^; CD105^+^/CD14^−^; CD19^−^; CD34^−^; CD45^−^) (MSC Phenotyping Kit; Miltenyi Biotec, Bergisch Gladbach, NRW, Germany) on flow cytometry (Novocyte 3000; ACEA Biosciences, San Diego, CA, USA) and sub-cultivated in DMEM (Biosera, Nuaille, France) supplemented with 10% FBS (Diagnovum, Ebsdorfergrund, Germany), 2 mM L-Glutamine (Sigma-Aldrich—Merck), 4.5 mg/mL D-Glucose (Sigma-Aldrich—Merck), 100 U/mL penicillin, 100 µg/mL streptomycin (Biosera) complete medium up to 4th passage prior cryopreservation experiments. For the ADSCs cell count and viability assessment electronic pulse area analysis and electronic current exclusion/pulse-field analysis were performed (CaSy cell counter, OMNI Life Science GmbH, Bremen, Germany).

### 2.3. Cryopreservation Protocol

Before cryopreservation, DPSCs/ADSCs of each lineage were divided into seven aliquots containing 0.5 × 10^6^ cells according to the CPA/s supplemented in the cryopreservation media (Table 1).

**Figure 1:**
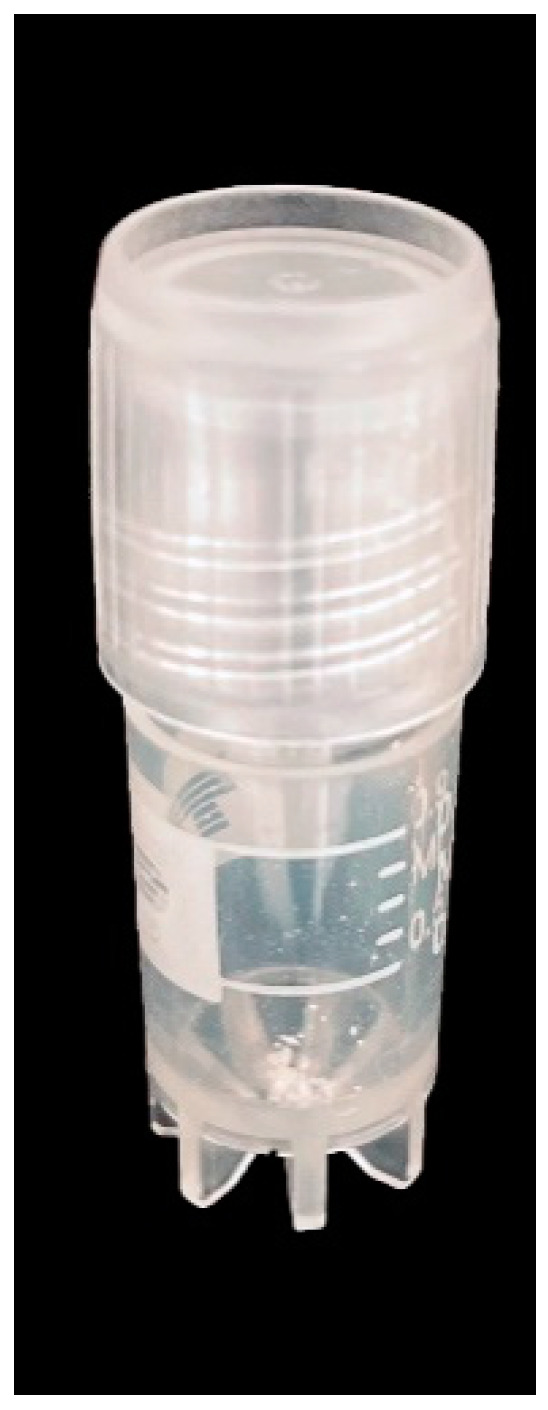
The preprepared cryovial containing a pharmacological grade high-molecular-weight HA (HMW-HA; >1.0 MDa).

The cryovials containing a pharmacological grade high-molecular-weight HA were distributed and preprepared by the company Contipro a.s., Dolni Dobrouc, Czech Republic (Figure 1).

First, we filled the experiment cryovials containing the HMW-HA with 0.5 mL of the modified cultivation media for mesenchymal adult progenitor cells minus the volume of cell aliquots containing 0.5 × 10^6^ cells submerged in the modified cultivation media. This was undertaken to dissolve any HMW-HA present before we placed the cells in each cryovial. Subsequently, we added the cell samples and 0.5 mL of precooled 4 °C cryopreservation medium composed of DMSO (Sigma-Aldrich—Merck KGaA) and FBS (PAA Laboratories) according to final concentrations of DMSO in the experiment groups, namely 5%, and 3%. In the control groups, we followed the same cryopreservation protocols but without the HMW-HA.

Afterward, we stored each cryovial using uncontrolled-rate freezing. The cryovials were placed at a temperature of −20 °C and kept for 1–1.5 h. Then, they were placed directly in the freezer and stored at −80 °C for one week.

### 2.4. hMSC Revival

After the one-week storage, each cryovial was thawed using a 37 °C thermal bath. The cells were mixed in a tube with an inactivation medium (α-MEM and FBS at a concentration of 20% for DPSCs; DMEM complete medium for ADSCs) to inactivate the cryopreservation medium and cryoprotectant/s. The tube was then centrifuged at 2000 rpm for 5 min (600× *g*).

### 2.5. Effect of Cryopreservation on DPSC Viability, Enumeration, and Cell Proliferation Activity

We measured the total cell count and viability in all groups after cryopreservation. We counted the total cell number using the Z2-Counter (Beckman Coulter), evaluated the viability using the trypan dye exclusion method along with a Vi-Cell analyzer (Beckman Coulter). Proliferation activity was determined as population doublings (PDs) and population doubling time (PDT). We used the formula PD = log_2_ (N_x_/N_1_) to calculate the population doublings. N_x_ is the total cell count calculated using the Z2-Counter upon cell cultivation, and N_1_ is the initial count of cryopreserved cells after thawing. To calculate the population doubling time, we used the formula PDT = t/n, where t is the number of hours of cultivation upon cell thawing, and n is the number of PDs, calculated as described above.

### 2.6. Effect of Cryopreservation on Phenotype Profile

The phenotype analysis was performed by first detaching adherent stem cells using the 0.05% trypsin-EDTA solution (Gibco) and then staining them with primary immunofluorescence antibodies conjugated with phycoerythrin (PE) or fluorescein (FITC). Positive cells were determined as the percentage with a fluorescence intensity greater than 99.5% of the negative isotype immunoglobulin control. We analyzed the following CD markers: CD29 (TS2/16, BioLegend, San Diego, CA, USA), CD31 (MBC 78.2, Invitrogen), CD34 (581 (Class 287 III, Invitrogen), CD44 (MEM 85, Invitrogen), CD45 (HI30, Invitrogen), CD49f (GoH3, Invitrogen), CD73 (AD2, BD Biosciences Pharmingen/Miltenyi Biotec), CD90 (F15-42-1-5, Beckman Coulter/Miltenyi Biotec).

### 2.7. Effect of Cryopreservation on Multipotency Capacity

For osteogenic differentiation, 70–100% confluent DPSCs were exposed to the Differentiation of Basal Medium-Osteogenic (Lonza, Basel, Switzerland) for three weeks. The differentiation medium was changed every third day of cultivation. At the end of the duration, induced cultures were evaluated for osteogenic differentiation using immunohistochemistry and histological staining. Osteogenic cultures were fixed using 10% formalin, embedded in paraffin, and cut in 7 µm sections. After deparaffinization, the sections were stained using von Kossa staining in order to expose calcium phosphate deposits (brown or black spots) and processed for anti-osteocalcin immunohistochemistry (rusty areas). Osteocalcin is one of the main proteins of the osteogenic extracellular matrix. For immunohistochemistry, sections were incubated with a primary antibody, a primary mouse IgG antibody (1:50, Millipore, Burlington, MA, USA), and a donkey anti-mouse secondary IgG antibody (1:250, Jackson ImmunoResearch Labs, West Grove, PA, USA). Chondrogenesis was initiated in 70–100% confluent DPSCs. Passaged DPSCs were trypsinized and resuspended in the Differentiation Basal Medium-Chondrogenic (Lonza); then supplemented with 50 ng/mL TGF-β1 (R&D Systems, Minneapolis, MN, USA) for three weeks. The chondrogenic medium was replenished twice a week. After three weeks, cultures were prepared for immunohistochemistry and histological staining in the same way as the osteogenic samples. Afterward, the sections were assessed with anti-type II collagen antibodies. Slices were incubated with a primary mouse IgM antibody (1:500, Sigma-Aldrich, Saint Louis, MO, USA) and Cy3TM-conjugated goat anti-mouse secondary IgM antibody. Cell nuclei were counterstained with 4′-6-diamidino-2-phenylindole (DAPI, Sigma-Aldrich). Besides the immunocytochemistry, the chondrogenic sections were stained with Alcian blue (for 30 min in 1% Alcian blue solution in 3% acetic acid) and Trichrome staining (modified according to Masson).

Due to the fact that even non-cryopreserved DPSC lineages differentiated into adipogenic cell lines unwillingly in our previous study [3], we did not induce adipogenesis in these DPSC groups.

### 2.8. Statistical Analysis

All statistical analyses were performed using the statistical software GraphPad Prism 6 (San Diego, CA, USA). The data are presented as the mean ± SD. The statistical significances (* *p* < 0.05) were calculated using either one-way ANOVA followed by Dunnett’s multiple comparison test for continuous variables, or Friedman’s test followed by Dunn’s multiple comparison test on ranks for nonparametric variables. The Shapiro–Wilk test or Kolmogorov–Smirnov test were used for normal distribution evaluations.

## 3. Results

### 3.1. Effect of Cryopreservation on DPSC Viability and Enumeration

The DPSCs were revived from all cryopreserved samples stored for one week using the uncontrolled-rate freezing technique and various concentrations of two CPAs (low and high molecular weight CPAs) or their combinations. The different strategies of the CPAs used copied the seven various cryopreservation groups (see Section 2).

**Figure 2:**
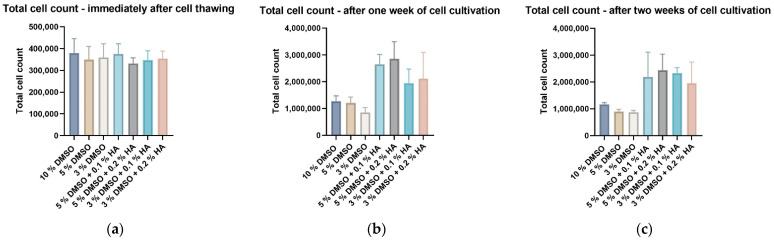
Total DPSC count measured using Z2-Counter (Beckman Coulter): (**a**) immediately after thawing; (**b**) after one week of cell cultivation; (**c**) after two weeks of cell cultivation. Data are presented as a mean and SD plotted as error bars. The statistical analysis was performed between control and experimental groups using Friedman’s test followed by Dunn’s multiple comparison test.

**Figure 3:**
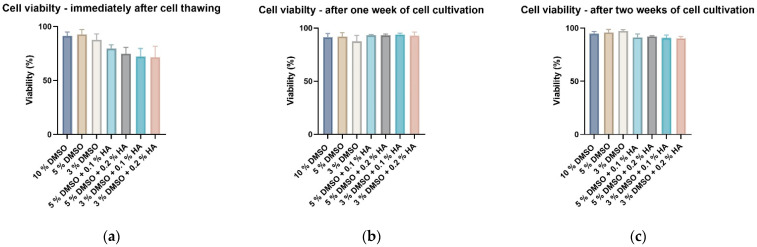
Viability of DPSCs measured using trypan blue exclusion method and the Vi-Cell analyzer (Beckman Coulter): (**a**) immediately after thawing; (**b**) after one week of cell cultivation; (**c**) after two weeks of cell cultivation. Data are presented as a mean and SD plotted as error bars. The statistical analysis was performed between control and experimental groups using Friedman’s test followed by Dunn’s multiple comparison test.

Immediately after thawing, the highest number of revived DPSCs with high cell viability was found in samples cryopreserved in standard 10% DMSO (Figure 2a and Figure 3a). In samples where the various concentrations of DMSO (as the LMW-CPA) and HA (as HMW-CPA) were used, the total cell count or cell viability (about 75% on average) was lower (Figure 2a and Figure 3a). However, the results became different after one or two weeks of cultivating the stored DPSCs (Figure 2b,c and Figure 3b,c). Cells stored using HMW-HA in combination with a lower (but effective) concentration of DMSO proliferated faster than the control groups cryopreserved without HMW-HA. Thus, after one week of cultivation, we observed approximately twice as many DPSCs in HA-cryopreserved samples than in controls of the same DMSO concentration. Moreover, the cell proliferation in samples stored with HA was upregulated even after two weeks of cultivation (Figure 3c). The cell viability accessed one and two weeks after sample revival was almost equal in all groups, control or experimental showing the minimal effect of cryopreservation on DPSC viability in this experimental setting.

We concluded that the combination of HMW-HA and DMSO protects DPSCs effectively during cryogenic storage using uncontrolled-rate freezing. The cryopreservation medium comprising 3% DMSO and 0.1% HMW-HA was identified as the most effective combination, regarding the fact that cell proliferation differences between this combination and its 3% DMSO control were the highest. Furthermore, this cryopreservation medium was with the lowest concentration of DMSO but also minimal HA supplementation. Therefore, we continued testing this combination in order to determine its effect on the DPSC phenotype profile and multipotency capacity upon cryopreservation.

### 3.2. Effect of Cryopreservation on Phenotype Profile

**Figure 4:**
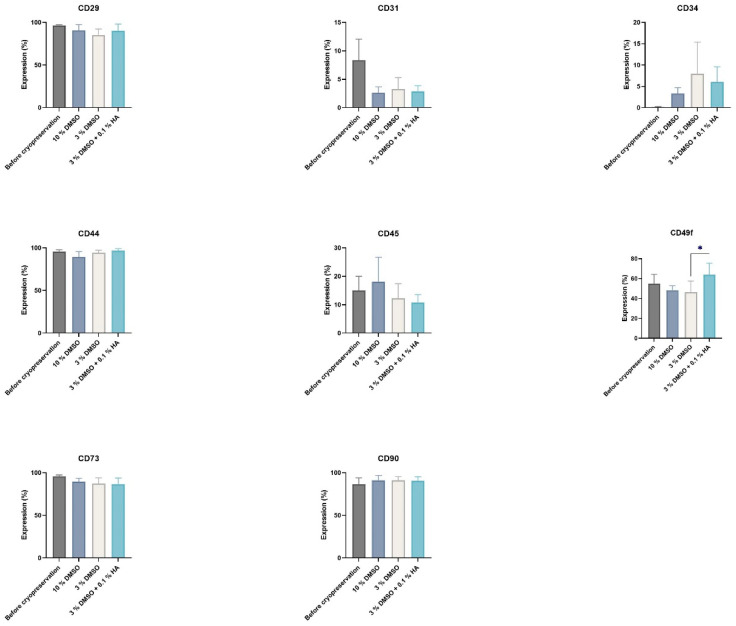
Phenotype profiles of DPSCs evaluated prior to and 2 weeks post-cryopreservation. The layouts of graphs illustrate percentages of positive cells, determined as the percentage with a fluorescence intensity greater than 99.5% of the negative isotype immunoglobulin control. Data are presented as a mean and SD plotted as error bars. The statistical significances (* *p* < 0.05) were calculated using either one-way ANOVA, followed by Dunnett’s multiple comparison test for continuous variables, or Friedman’s test, followed by Dunn’s multiple comparison test on ranks for nonparametric variables. The Shapiro–Wilk test or Kolmogorov–Smirnov test were used for normal distribution evaluations.

We studied the phenotype profile of DPSCs prior to and after cryopreservation and observed high expression of mesenchymal stem cell markers or stromal associated markers (CD29, CD44, CD73, CD90) and low or no expression of hematopoietic markers (CD34, CD45). The endothelial marker (CD31) was downregulated upon cryopreservation. Expression of CD49f marker was not changed upon cryopreservation in any of the control groups but was upregulated in samples cryopreserved by 3% DMSO + 0.1% HMW-HA cryomedium after two weeks of cultivation. The upregulated expression of CD49f corresponded to the increasing trend of DPSC proliferation in experimental groups where the HMW-HA was used in combination with DMSO (Figure 4).

### 3.3. Effect of Cryopreservation on Multipotency Capacity

**Figure 5:**
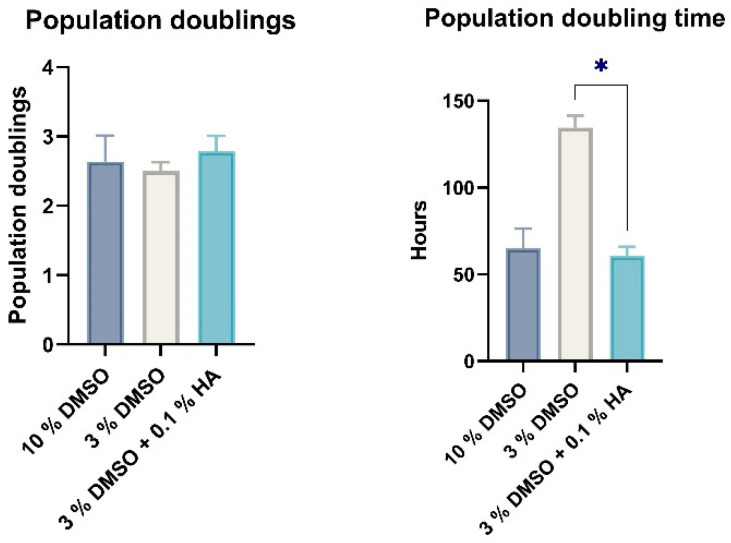
Population doublings and population doubling time of all cell groups reached from cell thawing to period of time when we induced differentiation. Data are presented as a mean and SD plotted as error bars. The statistical significances (* *p* < 0.05) were calculated using either one-way ANOVA, followed by Dunnett’s multiple comparison test for continuous variables, or Friedman’s test, followed by Dunn’s multiple comparison test on ranks for nonparametric variables. The Shapiro–Wilk test or Kolmogorov–Smirnov test were used for normal distribution evaluations.

**Figure 6:**
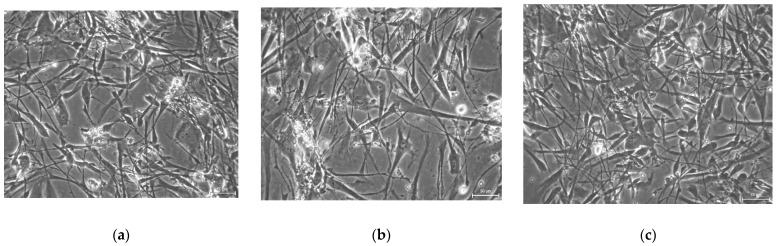
Revived DPSC before differentiation: (**a**) 10% DMSO cryomedium; (**b**) 3% DMSO cryomedium; (**c**) 3% DMSO + 0.1% HMW-HA cryomedium. Scale bar 50 μm.

Multipotency is one of the main features of stem cells, primarily in their potential usage in regenerative or reparative therapies. To determine the effect of three various CPAs on multipotency capacity we revived DPSCs after one-week cryogenic storage at −80 °C. We reseeded DPSCs from all groups in the culture dishes and cultivated them until they reached approximately 70–100% confluence (Figure 2). However, DPSCs stored using 3% DMSO as the CPA with no HMW-HA had lower proliferation activity in comparison with 10% DMSO cryomedium and 3% DMSO with 0.1% HMW-HA, respectively; thus they required a prolonged period of cultivation to get optimal confluency for multipotency assessment (Figure 5 and Figure 6a–c).

**Figure 7:**
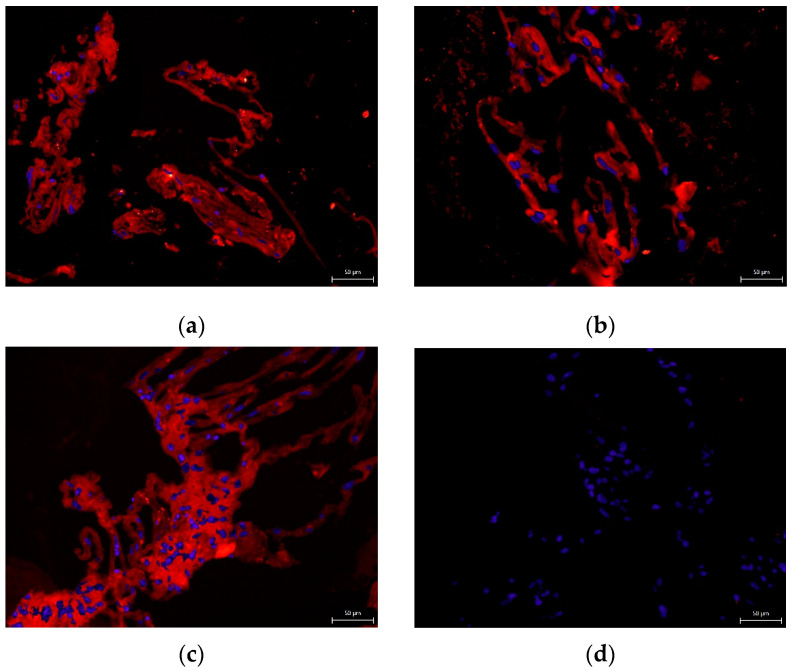
Immunocytochemical detection of type II collagen in the extracellular chondrogenic mass of the DPSCs after 3-week cultivation in a chondrogenic differentiation medium. Type II collagen shows up as fluorescent red, and stem cell nuclei fluorescent blue: (**a**) 10% DMSO cryomedium; (**b**) 3% DMSO cryomedium; (**c**) 3% DMSO + 0.1% HMW-HA cryomedium; (**d**) non-differentiated DPSCs. Scale bar 50 μm.

**Figure 8:**
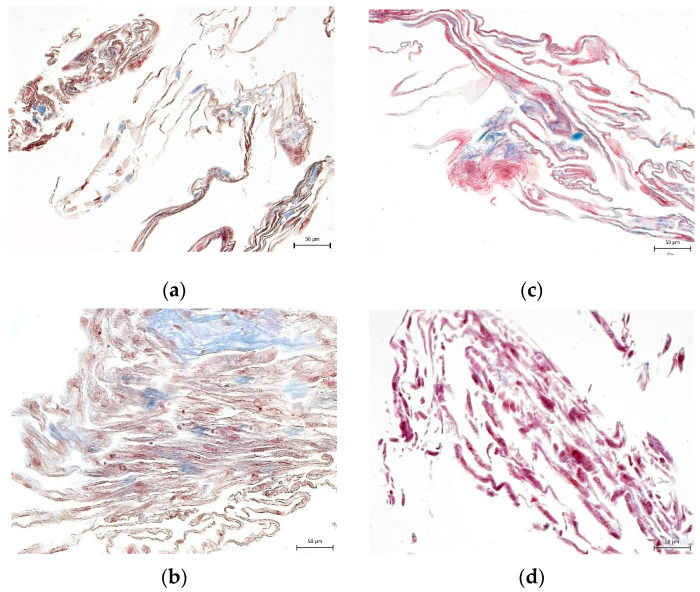
Detection of collagen and procollagen in the extracellular chondrogenic mass of DPSCs after 3-week cultivation in a chondrogenic differentiation medium. After histological staining using blue Masson’s trichrome, the collagen and procollagen appear as blue areas: (**a**) 10% DMSO cryomedium; (**b**) 3% DMSO cryomedium; (**c**) 3% DMSO + 0.1% HMW-HA cryomedium; (**d**) non-differentiated DPSCs. Scale bar 50 μm.

**Figure 9:**
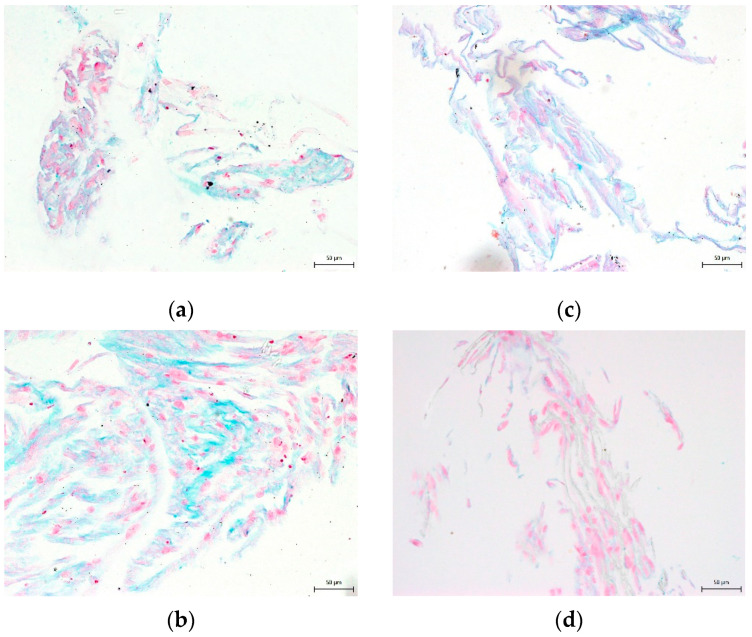
Detection of acid mucopolysaccharides in the extracellular chondrogenic mass of DPSCs after 3-week cultivation in a chondrogenic differentiation medium. After histological staining using Alcian blue, the acid mucopolysaccharides appear as turquoise areas: (**a**) 10% DMSO cryomedium; (**b**) 3% DMSO cryomedium; (**c**) 3% DMSO + 0.1% HMW-HA cryomedium; (**d**) non-differentiated DPSCs. Scale bar 50 μm.

**Figure 10:**
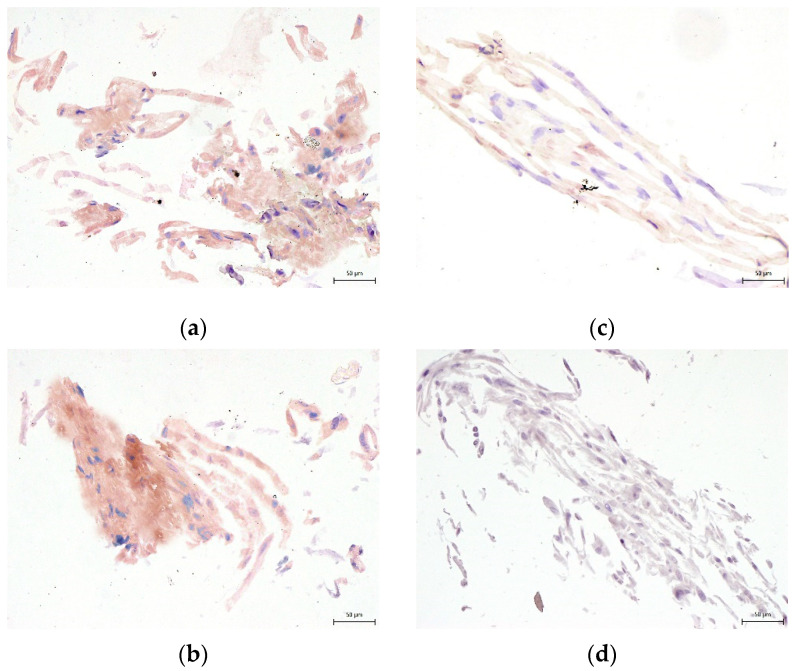
Immunocytochemical detection of osteocalcin, one of the main proteins of the extracellular osteogenic matrix. Samples of DPSCs are after 3-week cultivation in osteogenic differentiation medium. Osteocalcin is revealed as brown areas: (**a**) 10% DMSO cryomedium; (**b**) 3% DMSO cryomedium; (**c**) 3% DMSO + 0.1% HMW-HA cryomedium; (**d**) non-differentiated DPSCs. Scale bar 50 μm.

**Figure 11:**
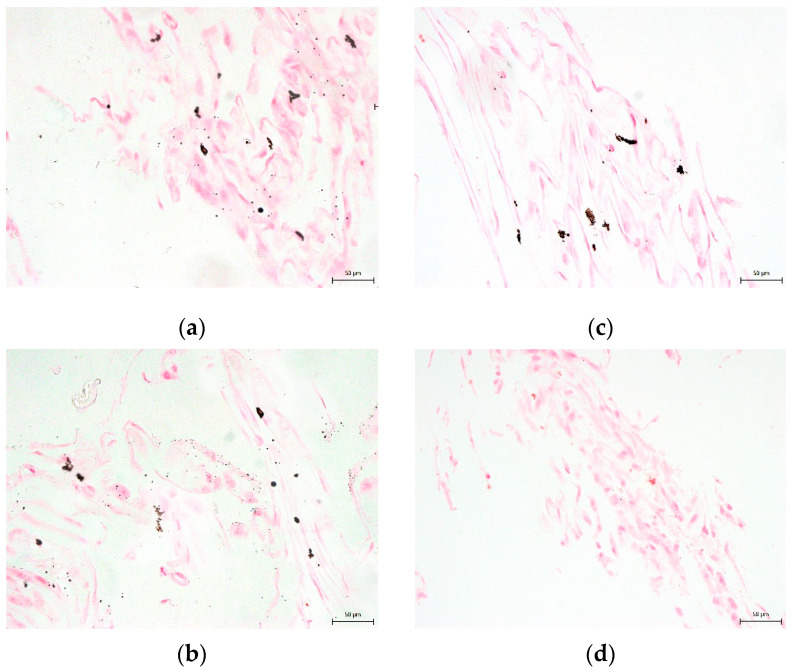
The presence of calcium phosphate deposits in the extracellular osteogenic matrix. Samples of lineage DPSCs are after 3-week cultivation in osteogenic differentiation medium. Calcium phosphate deposits are colored as brown–black areas: (**a**) 10% DMSO cryomedium; (**b**) 3% DMSO cryomedium; (**c**) 3% DMSO + 0.1% HMW-HA cryomedium; (**d**) non-differentiated DPSCs. Scale bar 50 μm.

None of the cryomedium compositions negatively affected the differential potential of DPSC into chondrogenic and osteogenic cell lineages. To determine multipotency capacity we performed immunohistochemistry staining to reveal type II collagen in chondrogenic differentiated DPSCs (Figure 7a–d) and osteocalcin in osteogenic differentiated DPSCs (Figure 10a–d). We also stained cells after induced differentiation using histological staining (Figures 8a–d, 9a–d and 11a–d). The following Figure 7, Figure 8, Figure 9, Figure 10 and Figure 11 illustrate that we were able to trigger osteogenesis and chondrogenesis with no difference among the cryopreservation medium.

### 3.4. Robustness of HA Cryoprotection Effectivity on ADSC Cryopreservation

**Figure 12:**
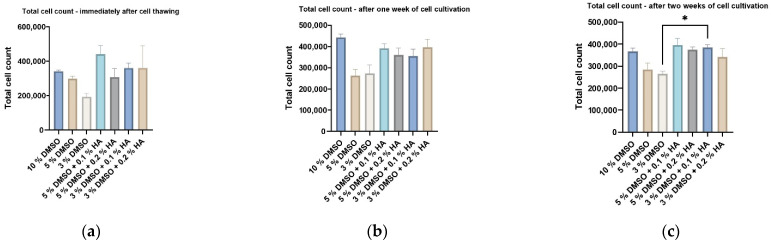
Total ADSCs count measured using CaSy cell counter (OMNI Life Science GmbH): (**a**) immediately after thawing; (**b**) after one week of cell cultivation; (**c**) after two weeks of cell cultivation. Data are presented as a mean and SD plotted as error bars. The statistical significances (* *p* < 0.05) were determined between control and experimental groups using Friedman’s test followed by Dunn’s multiple comparison test.

**Figure 13:**
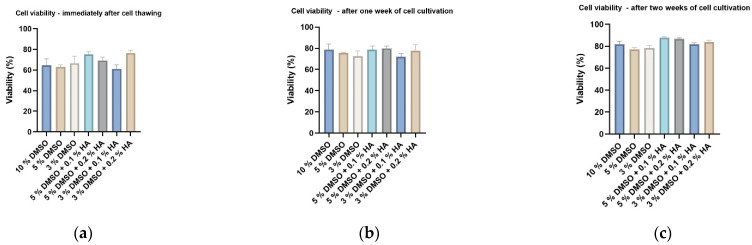
Viability of ADSCs measured using pulse area analysis and electronic current exclusion/pulse-field analysis on CaSy cell counter (OMNI Life Science GmbH): (**a**) immediately after thawing; (**b**) after one week of cell cultivation; (**c**) after two weeks of cell cultivation. Data are presented as a mean and SD plotted as error bars. The statistical analysis was performed between control and experimental groups using Friedman’s test followed by Dunn’s multiple comparison test.

To test whether observed effects of HMW-HA on cryopreservation effectivity were not DPSC-specific, we cryopreserved different subtypes of hMSC than DPSC—the adipose-tissue derived stem cells (hADSCs). hADSCs were cryopreserved by six groups of cryoprotective media according to combinations previously described on DPSCs. Decreased DMSO concentration to 5 and 3% had the consequence of affected survival of ADSCs compared to standard conditions of 10% DMSO immediately after cell thawing (Figure 12a). One week after cell revival, the reduced cell count became more prominent. Moreover, reduced total cell count was observed up to two weeks of cell cultivation (Figure 12b,c). Enrichment of 5 and 3% DMSO cryomedium with HMW-HA of two concentrations—0.1 and 0.2%—led to a rescue effect in total cell count. The first week after cryopreservation, cell count with reduced DMSO concentration but supplemented by 0.1% HA overgrew its control and reached values of standard, which was reflected also after two weeks of cultivation Figure 12b,c). As the most effective 3% DMSO augmented by 0.1% HA was identified, since cell count difference between this combination and its 3% DMSO control was significantly highest and simultaneously the concentration of both DMSO and HA was lowest (Figure 12c). The cell viability was not significantly changed neither by decreased DMSO concentration nor HMW-HA enrichment in any of the experimental time points (Figure 13a–c). Those data confirmed the cryoprotection effect of HMW-HA previously seen in DPSCs cryopreservation.

**Figure 14:**
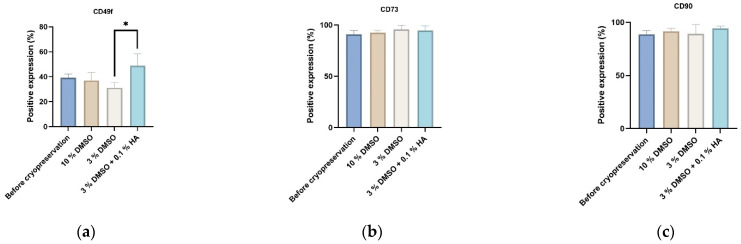
Phenotype profiles of ADSCs, evaluated after and prior to and 2 weeks post-cryopreservation. The layouts of graphs illustrate percentages of positive cells, determined as the percentage with a fluorescence intensity greater than 99.5% of the negative isotype immunoglobulin control. Data are presented as a mean and SD plotted as error bars. The statistical significances (* *p* < 0.05) were calculated using one-way ANOVA, followed by Dunnett’s multiple comparison test for continuous variables.

To identify the potential effect of cryopreservation on stemness of the ADSCs, we accessed surface stem cell phenotype markers prior to and after cryopreservation (Figure 14a–c). Similarly like with DPSCs cryopreservation, we observed a significant increase in stemness marker CD49f on ADSC cell lines cryopreserved in 3% DMSO + 0.1% HMW-HA after two weeks post-cryopreservation in comparison to their controls (Figure 14a). Expression of stem cell markers CD73 and CD90 was only marginally modulated after cryopreservation independently of decreased DMSO content or HA enrichment (Figure 14b,c).

Presented results indicated high effectivity of HMW-HA supplementation on hMSC survival, proliferation, and maintenance of stem cell phenotype over 1 week of cryopreservation. To determine the effect of prolonged cryostorage we performed comparative experiments consisting of two weeks and two months of storage at −80 °C (Supplementary Information Figures S1–S3). We observed a similar effect in 3% DMSO + 0.1% HMW-HA cryopreserved samples after one and two weeks of cell cultivation following two weeks/months of cryopreservation on cell survival (Figure S1a) and proliferation (Figure S1b,c), as well as sustained high viability of the ADSCs after cryopreservation (Figure S2a–c). A significant increase in stemness marker CD49f on ADSC cell lines cryopreserved in 3% DMSO + 0.1% HMW-HA was also presented after two weeks and two months of cryostorage (Figure S3a), and high expression of CD73 and CD90 was maintained (Figure S3b,c).

## 4. Discussion

Cryopreservation represents an efficient method for preserving and pooling mesenchymal stem cells to obtain the cell counts required for regenerative or reparative therapies and facilitate its off-the-shelf use. Due to risks connected with long-term stem cell cultivation, such as contamination, genetic drifts, or epigenetic changes, it is necessary to store stem cells in unaltered form, if possible. Equally, during cryopreservation, it is important to preserve MSC functional properties such as immunomodulatory properties and multilineage differentiation ability.

However, cryopreservation also involves issues related to the higher risk of freeze injury and subsequent cell death due to the formation of intra- or extracellular ice crystals and the increase in solutes when cell suspension is cooled below the freezing point. In order to address this issue, CPAs are necessary to minimize or prevent the damage associated with the freezing process. The mechanisms of protection are not exactly understood. However, CPAs work primarily to alter the physical conditions of both ice and the solutions surrounding cells or the intracellular environment. To provide optimal protection, different CPAs have been identified [11,14,27]. Optimization of the use of CPA is one of the remaining challenges to be addressed in order to preserve MSCs effectively.

DMSO with a concentration of 10% combined with FBS is often used to preserve MSCs [8,14,28,29]. Thanks to its low molecular weight, DMSO can penetrate cells, depress the freezing point, and form extensive hydrogen bonds with water molecules; thus potentially altering the water-to-ice transition [29,30,31]. Despite its cryoprotective advantages, DMSO is not completely inert, and its cytotoxic effect increases with temperature, concentration, and exposure time [32]. Concerns over the negative effect of DMSO may be alleviated by lowering the dose of DMSO (from 10% to 5% [21] or 3.5% [33]), washing cells to remove DMSO before their application [34], or usage of a magnetic field during cryopreservation [22]. However, the decrement of DMSO content via expensive and laborious physical processes represents an important disadvantage for research or clinical laboratory application. The introduction of non-penetrating CPAs into the cryogenic media might be also one of the mechanisms to reduce the concentration of DMSO. High-molecular weight CPAs cannot cross the cell membrane but can protect the cells from rupture by forming a viscous glassy shell around the outer surface of the cells [35]. However, such CPAs are less efficient than DMSO in terms of maintaining the survival rate of revived stem cells. Therefore, they are mainly supplemented in the cryopreservation medium in order to lower the dose of DMSO. The optimal concentration of these cryoprotective agent “cocktails” as an alternative to DMSO alone has to be determined experimentally for each cell type.

According to the results, we demonstrated that the HMW-HA (>1.0 MDa) can be used as a cryoprotectant in the cryopreservation of DPSCs; thus reducing the concentration of DMSO. HA is the most versatile macromolecule present in the connective tissues of all vertebrates. With its excellent physicochemical properties (such as biodegradability, biocompatibility, nontoxicity, and no immunogenicity), hyaluronic acid has a wide range of applications and serves as an excellent tool in biomedical applications [36]. It is also a powerful antioxidant [37] and is best known for its ability to bond to water [36,38]. Therefore, our hypothesis was that oxidative damage and ice crystal formation during cryopreservation may be reduced by adding HA in the freezing medium. Other authors had already demonstrated, for example, that the quality of post-thaw sperm was higher when HA was added to freezing media [39].

We tested 0.1% and 0.2% HMW-HA in combination with reduced concentrations of DMSO (5% and 3%). hMSCs stored using 10%, 5%, and 3% DMSO served as control. DMSO concentrations below 2% were reposted as ineffective in the protection of cryopreserved stem cells [21]. Samples cryopreserved with supplementation of HMW-HA had increased proliferation after one or two weeks of cultivation in comparison with controls cryopreserved without HMW-HA, and cell viability was almost equal during subsequent cultivation independent of the CPA used during cryopreservation. The observed effect on cell survival and proliferation was universal among different hMSC sources, both DPSCs and ADSCs cryopreserved with supplementation of HMW-HA exceeded its controls. The increase in total cell count corresponded to the elevated expression of CD49f in samples cryopreserved with HMW-HA supplementation. Besides these biological markers for mesenchymal stem cells, other cell-surface proteins, including integrin α6, CD49f, have been used to characterize MSCs [40]. CD49f belongs to the group of type I integrins (transmembrane glycoproteins) that play a role in cell adhesion and signaling. Their activation affects signal transduction, cell differentiation, proliferation, cell shape, and survival [41]. Integrin α6 is also involved in maintaining stem cell self-renewal potential [41] and therefore can be considered a “stemness” marker. Moreover, none of the cryomedium compositions negatively affected the differential potential of DPSCs into chondrogenic and osteogenic cell lineages. Thus, chondrogenesis and osteogenesis DPSCs differentiation has been observed independently of the CPA used during cryopreservation. We did not induce adipogenesis in any group of cells since in our previous study we were not able to induce even non-cryopreserved cells to differentiate into adipocytes [3]. These results correspond with outcomes published by Gronthos. He concluded that DPSCs generally differentiate in adipocytes unwillingly [1].

To sum all up, in samples cryopreserved with the combination of 3% DMSO + 0.1% HMW-HA, we observed a high post-thaw recovery of MSCs in subsequent cultivation. MSCs stored using this cryopreservation medium highly proliferated, kept the high viability, demonstrated an immunoprofile of mesenchymal stem cells with the highest increase level of CD49f, and have unchanged differentiation capacity. Furthermore, this CPA “cocktail” contains the lowest dose of DMSO and minimal HA supplementation.

The hyaluronic acid of biotechnology origin represents an effective animal component-free cryoprotective compound. That is advantageous for producing the potential ready-to-use product used as CPA in cryopreservation of mesenchymal stem cells for clinical use of MSCs in human medicine. Utilization of a pharmacological grade high-molecular-weight HA in cryopreservation of stem cells can be an example of a process that falls within the concept of biotechnology. Today, biotechnology covers many different disciplines and belongs to rapidly growing fields. New technologies and products are developed every year. We will be more than satisfied if our results will participate in producing the new product and help to decrease the DMSO concentration in the cryogenic medium for mesenchymal stem cells.

## 5. Conclusions

To our knowledge, our study is the first to evaluate the effect of HMW-HA when used as a CPA during uncontrolled-rate freezing of DPSCs. Supplementing HMW-HA into the cryopreservation medium allowed us to reduce DMSO concentrations from 10% to 3% with no effect on viability or mesenchymal stem cell markers. DPSCs stored using 3% DMSO and 0.1% HMW-HA proliferated faster than DPSCs stored with no HMW-HA supplement. We gained more DPSCs after two weeks of cultivation in comparison with DPSCs cryopreserved using 10%, 5%, or 3% DMSO alone. The proliferation increase corresponded with an elevated expression of stemness marker CD49f. The observed effect on cell survival and proliferation was universal among different hMSC sources. Thus, we conclude that HA facilitates a substantial decrease in potentially harmful DMSO in cryopreservation medium to minimal content without any consequences on hMSC survival, proliferation, and stemness. Our results will lead to producing the ready-to-use product that will be available on the market as CPA for cryopreservation of mesenchymal stem cells.

## 6. Patents

The action of HA in the stem cell cryopreservation is the subject of the following patent application by Contipro a.s. (Name: Hyaluronic acid containing cryopreservation medium, its use and form of cryopreservation, Application Number: PV 2022-148, Filed on: 8 April 2022). This does not alter the authors’ adherence to all MDPI policies on sharing data and materials.

## Figures and Tables

**Table 1:** Overview of CPA specifications used during uncontrolled-rate freezing of DPSCs.

Cryoprotective Agent/s
10% DMSO
5% DMSO
3% DMSO
5% DMSO + 0.1% HA
5% DMSO + 0.2% HA
3% DMSO + 0.1% HA
3% DMSO + 0.2% HA

## Data Availability

Data is contained within the article.

## Supplementary Materials

The following supporting information can be downloaded at: https://www.mdpi.com/article/10.3390/biom12050610/s1, Figure S1: Comparison of 2 weeks/2months cryostorage. Total ADSCs count measured using CaSy cell counter (OMNI Life Science GmbH): (a) immediately after thawing; (b) after one week of cell cultivation; (c) after two weeks of cell cultivation. Data are presented as a mean and SD plotted as error bars. The statistical significances (* *p* < 0.05) were performed between control and experimental groups using Friedman’s test followed by Dunn’s multiple comparison test, Figure S2: Comparison of 2 weeks/2months cryostorage. Viability of ADSCs measured using pulse area analysis and electronic current exclusion/pulse-field analysis on CaSy cell counter (OMNI Life Science GmbH): (a) immediately after thawing; (b) after one week of cell cultivation; (c) after two weeks of cell cultivation. Data are presented as a mean and SD plotted as error bars. The statistical analysis was performed between control and experimental groups using Friedman’s test followed by Dunn’s multiple comparison test, Figure S3: Comparison of 2 weeks/2months cryostorage. Phenotype profiles of ADSCs, evaluated after and prior to and 2 weeks post-cryopreservation. The layouts of graphs illustrate percentages of positive cells, determined as the percentage with a fluorescence intensity greater than 99.5% of the negative isotype immunoglobulin control. Data are presented as a mean and SD plotted as error bars. The statistical significances (* *p* < 0.05; ** *p* < 0.01) were calculated using one-way ANOVA, followed by Dunnett’s multiple comparison test for continuous variables.
