# Supplementary material for: Innovative Approach in the Cryogenic Freezing Medium for Mesenchymal Stem Cells
Source: Biomolecules. 2022 Apr 20;12(5):610. doi: 10.3390/biom12050610 (PMC9138570; doi:10.3390/biom12050610)
Supplement: Supplementary file 1 [file biomolecules-12-00610-s001.zip › biomolecules-1697099-supplementary.pdf]

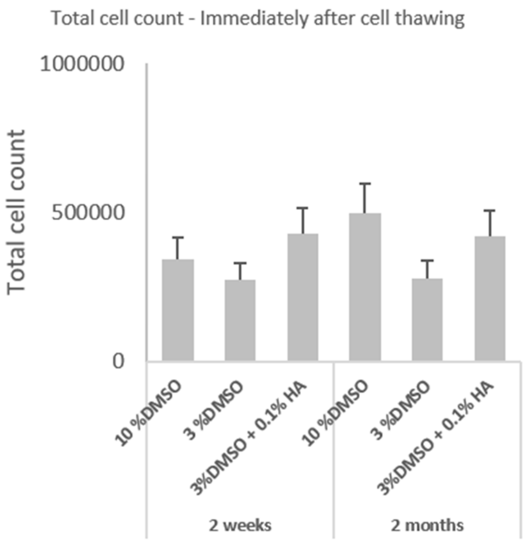

(**a**)

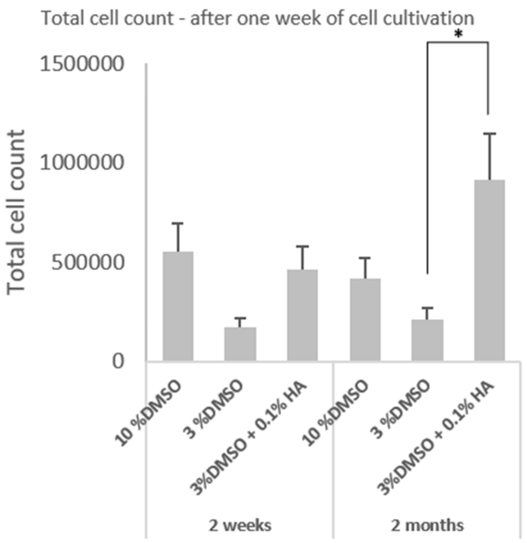

(**b**)

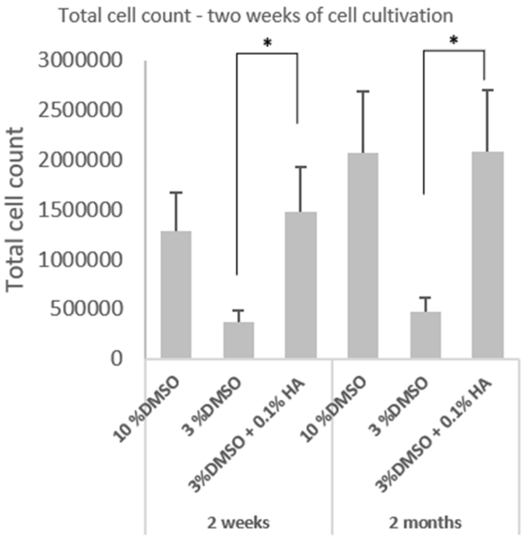

(**c**)

Figure S1. Comparison of 2 weeks/2months cryostorage. Total ADSCs count measured using CaSy cell counter (OMNI Life Science GmbH): (**a**) immediately after thawing; (**b**) after one week of cell cultivation; (**c**) after two weeks of cell cultivation. Data are presented as a mean and SD plotted as error bars. The statistical significances (* $p < 0.05$) were performed between control and experimental groups using Friedman's test followed by Dunn's multiple comparison test.

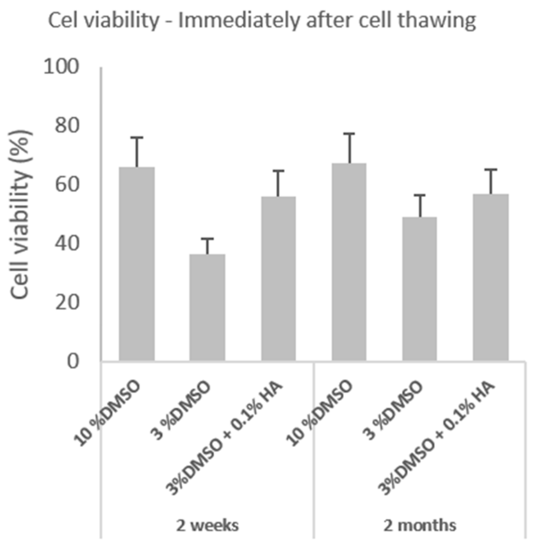

(**a**)

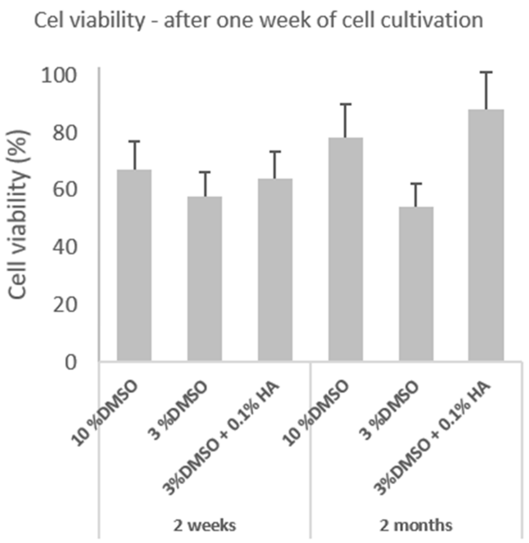

(**b**)

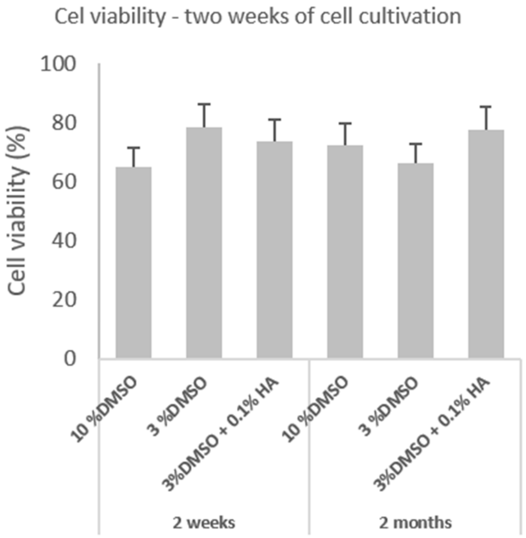

(**c**)

Figure S2. Comparison of 2 weeks/2months cryostorage. Viability of ADSCs measured using pulse area analysis and electronic current exclusion/pulse-field analysis on CaSy cell counter (OMNI Life Science GmbH): (**a**) immediately after thawing; (**b**) after one week of cell cultivation; (**c**) after two weeks of cell cultivation. Data are presented as a mean and SD plotted as error bars. The statistical analysis was performed between control and experimental groups using Friedman's test followed by Dunn's multiple comparison test.

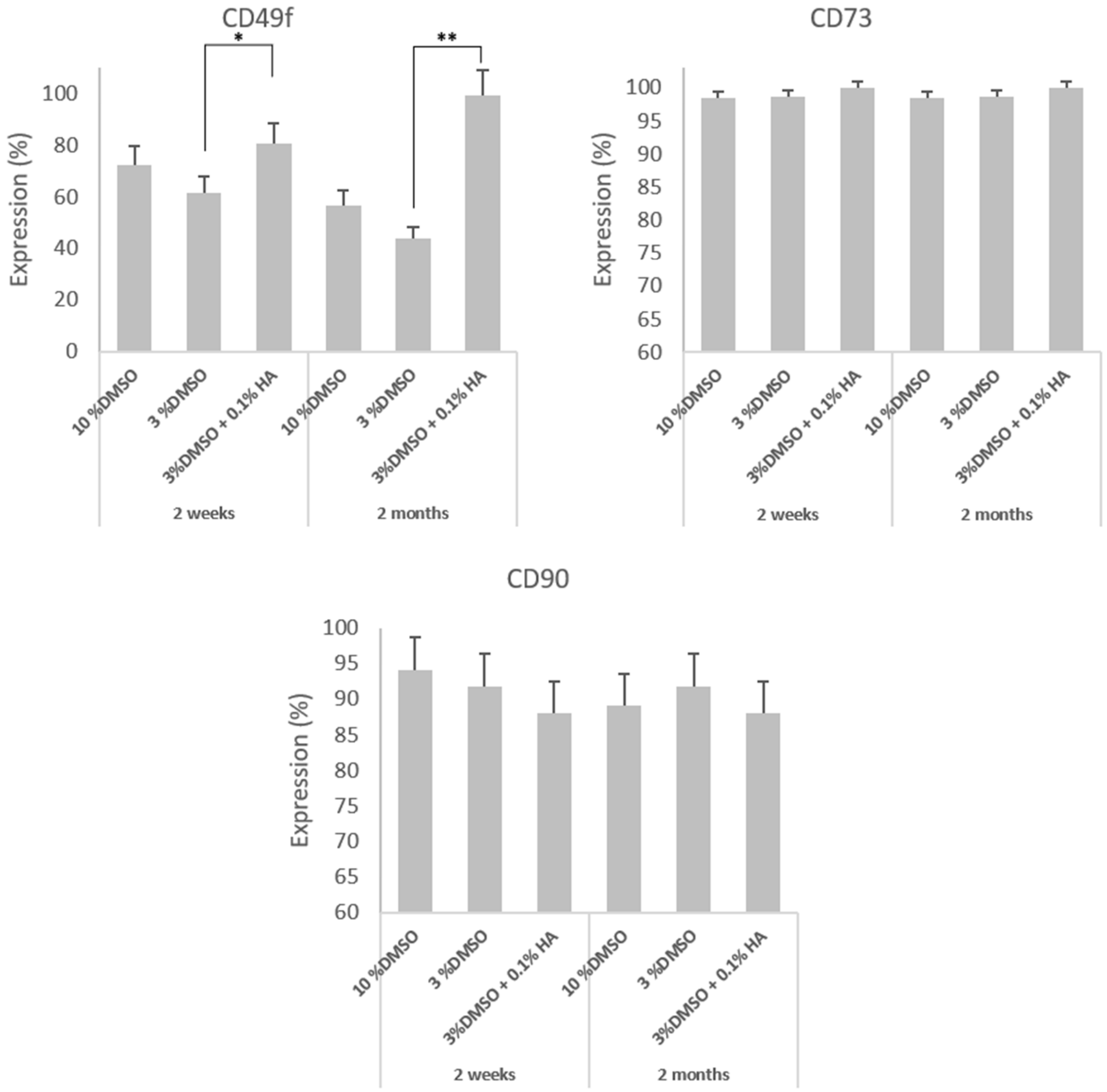

Figure S3. Comparison of 2 weeks/2months cryostorage. Phenotype profiles of ADSCs, evaluated after and prior to and 2 weeks post-cryopreservation. The layouts of graphs illustrate percentages of positive cells, determined as the percentage with a fluorescence intensity greater than 99.5% of the negative isotype immunoglobulin control. Data are presented as a mean and SD plotted as error bars. The statistical significances (* $p < 0.05$; ** $p < 0.01$) were calculated using one-way ANOVA, followed by Dunnett's multiple comparison test for continuous variables.
